# Contribution of glutamate decarboxylase in *Lactobacillus reuteri* to acid resistance and persistence in sourdough fermentation

**DOI:** 10.1186/1475-2859-10-S1-S8

**Published:** 2011-08-30

**Authors:** Marcia S Su, Sabine Schlicht, Michael G Gänzle

**Affiliations:** 1 University of Alberta, Department of Agricultural, Food and Nutritional Science, Edmonton, Canada

## Abstract

**Background:**

Acid stress impacts the persistence of lactobacilli in industrial sourdough fermentations, and in intestinal ecosystems. However, the contribution of glutamate to acid resistance in lactobacilli has not been demonstrated experimentally, and evidence for the contribution of acid resistance to the competitiveness of lactobacilli in sourdough is lacking. It was therefore the aim of this study to investigate the ecological role of glutamate decarboxylase in *L. reuteri*.

**Results:**

A gene coding for a putative glutamate decarboxylase, *gadB*, was identified in the genome of *L. reuteri* 100-23. Different from the organization of genetic loci coding for glutamate decarboxylase in other lactic acid bacteria, *gadB* was located adjacent to a putative glutaminase gene, *gls3.* An isogenic deletion mutant, *L. reuteri* ∆*gadB*, was generated by a double crossover method. *L. reuteri* 100-23 but not *L. reuteri ∆gadB* converted glutamate to γ-aminobutyrate (GABA) in phosphate butter (pH 2.5). In sourdough, both strains converted glutamine to glutamate but only *L. reuteri* 100-23 accumulated GABA. Glutamate addition to phosphate buffer, pH 2.5, improved survival of *L. reuteri* 100-23 100-fold. However, survival of *L. reuteri ∆gadB* remained essentially unchanged. The disruption of *gadB* did not affect growth of *L. reuteri* in mMRS or in sourdough. However, the wild type strain *L. reuteri* 100-23 displaced *L. reuteri ∆gadB* after 5 cycles of fermentation in back-slopped sourdough fermentations.

**Conclusions:**

The conversion of glutamate to GABA by *L. reuteri* 100-23 contributes to acid resistance and to competitiveness in industrial sourdough fermentations. The organization of the gene cluster for glutamate conversion, and the availability of amino acids in cereals imply that glutamine rather than glutamate functions as the substrate for GABA formation. The exceptional coupling of glutamine deamidation to glutamate decarboxylation in *L.Â reuteri* likely reflects adaptation to cereal substrates.

## Background

Sourdough is used in bread production as a leavening agent, or as a baking improver to improve the texture, flavour, and shelf life of bread [1,2]. Sourdoughs used as the sole leavening agent (type I sourdoughs) are maintained by frequent back-slopping. These conditions select for fast growing microorganisms, and type I sourdough microbiota are typically dominated by *Lactobacillus sanfranciscensis*[3-5]. Industrial sourdoughs used for the production of baking improvers (type II sourdoughs) are fermented at 35 – 40°C for extended fermentation times (2 – 5 days) to achieve high levels of total titrable acidity [1,3]. These sourdoughs are typically dominated by thermophilic, acid-tolerant lactobacilli, including *Lactobacillus pontis*, and *Lactobacillus panis*[3-5]. *Lactobacillus reuteri* is generally considered an intestinal organism [6] but also occurs in type II sourdoughs [3-5], and was shown to persist in an industrial sourdough fermentation over a period of more than one decade, corresponding to several 10,000 generations of bacterial growth [7]. Model sourdough fermentations confirmed that long fermentation times at high temperature (42°C) select for *L. reuteri*[8,9].

Owing to long fermentation times, acid stress impacts the persistence of lactobacilli in type II sourdough fermentation. Mechanisms of acid tolerance in Gram-positive bacteria generally include the expression of stress proteins, the overexpression of proton pumps, and modification of metabolic pathways to consume protons [10,11]. Remarkably, metabolic pathways contributing to acid resistance of *L. reuteri* also improve bread quality. The formation of dextran, reuteran, or levan by lactic acid bacteria contributes to acid resistance [12,13] and improves bread texture and volume [14]. Conversion of arginine to ornithine improves the acid resistance of *L. reuteri*[10,15,16]. Ornithine is also a precursor of the character impact compound of wheat bread crust flavour, 2-acetyl-1-pyrroline [17]. The deamidation of glutamine to glutamate by sourdough lactobacilli generates umami taste in sourdough bread, and improves growth of *L. reuteri* at low pH [18].

Glutamate decarboxylation to γ-aminobutyric acid (GABA) contributes to the acid resistance of *Escherichia coli*, *Listeria monocytogenes*, and *Lactococcus lactis*[19-21]. The antiport of glutamate and GABA generates a ΔpH and ΔΨ contributing to a proton motive force for ATP synthesis [22]. However, although glutamate decarboxylases were biochemically characterized in *L. brevis* and *L. paracasei*[23,24], the physiological function of glutamate decarboxylase in aciduric *Lactobacillus* is unclear. Moreover, experimental evidence for the contribution of any acid resistance mechanism to the competitiveness of lactobacilli in sourdough is lacking. It was therefore the aim of this study to investigate the ecological role of glutamate decarboxylase in cereal-associated *L. reuteri*. Experiments were conducted with *L. reuteri* 100-23 [25]. This strain has a known genome sequence, harbours a glutamate decarboxylase, and decarboxylates glutamate during sourdough fermentation [26]. An isogenic deletion mutant, *L. reuteri* ∆*gadB*, was generated by a double crossover method to elucidate the importance of GadB in acid resistance and the competitiveness in type II sourdoughs.

## Methods

### Strains, plasmids and media

Bacterial strains and plasmids used in this study are shown in Table 1. *Escherichia coli* JM109 (Promega, Nepean, Canada) was cultured in Luria-Bertani (LB) broth at 37°C or 30°C. *L. reuteri* was cultured at 37°C in deMan-Rogosa-Sharpe broth (MRS) (Difco, Mississauga, Canada) or modified MRS medium [7]. Ampicillin (100 mg L^-1^) or erythromycin (500 mg L^-1^) was added to LB for selecting antibiotic-resistant *E. coli*. Erythromycin (10 mg L^-1^) was added to MRS medium to select erythromycin-resistant *L. reuteri.*

**Table 1:** Bacterial strains and plasmids used in this study

Strain or plasmid	Genotype	Source or reference
**Strains**		
*Escherichia coli* JM109	Cloning host for pGEMTeasy- and pJRS233-derivative plasmids	Promega
*Lactobacillus reuteri* 100-23	Rodent isolate; wild type strain	[25]
Δ*gadB*	Wild-type strain derivative with a deletion in *gadB*	This study
**Plasmids**		
pGEMTeasy	Cloning vector used in *E. coli*; 3.0 kb; Amp^r^	Promega
pGadB-A	pGEMTeasy containing 1 kb of the DNA sequence upstream of *gadB*; 4.0 kb; Amp^r^	This study
pGadB-B	pGEMTeasy containing 0.9 kb of the DNA sequence downstream of *gadB*; 3.9 kb; Amp^r^	This study
pGadB-AB	pGEMTeasy containing the upstream and downstream sequences of *gadB*; 4.9 kb; Amp^r^	This study
pJRS233	Shuttle vector used in the hosts *E. coli* and *L. reuteri* 100-23; 6.0 kb; Erm^r^	[29]
pKO-*gadB*-AB	pJRS233 containing 1.9 kb of the flanking sequences of *gadB*; 7.9 kb; Erm^r^	This study

Amp^r^: amplicillin-resistance gene; Erm^r^: erythromycin-resistance gene

### DNA manipulation

DNA was isolated from overnight cultures using the Blood & Tissue Kit (Qiagen, Mississauga, Canada) according to instructions of the manufacturer. Oligonucleotides were purchased from Integrated DNA Technologies (San Diego, CA), restriction enzymes from New England Biolabs (Pickering, Canada), T4 DNA ligase from Epicentre (Markham, Canada) and Taq DNA polymerase from Invitrogen (Burlington, Canada). DNA sequencing was performed by Macrogen (Rockville, Maryland).

### Amino acid comparison of *gadB* genes and the gene loci in lactic acid bacteria

The glutamate decarboxylase (GadB) protein sequence of *Lactobacillus paracasei*[24] (GI:169264609) was used to identify a homologous gene encoding *gadB* in the genome of *L. reuteri* 100-23 (http://www.jgi.doe.gov/) [27]. Gene loci of putative *gadB* genes and flanking nucleotide sequences in *Lactococcus lactis* subsp. *cremoris* MG1363 [21], *L. reuteri* 100-23 and *L. plantarum* WCFS1 [28] were analyzed with the BLASTx program against the National Center for Biotechnology Information databases (http://blast.ncbi.nlm.nih.gov/Blast.cgi). The protein sequences of GadB from various species were retrieved from Uniprot database and aligned to calculate the score of similarity using Geneious alignment (Geneious version 5.1.6, http://www.geneious.com).

### Generation and verification of the *L. reuteri* Δ*gadB* mutant

The gene coding for GadB in *L. reuteri* 100-23 was truncated using pJRS233 [29] according to a deletion strategy described earlier [30]. A 5’ 1016-bp fragment of *gadB* was amplified from genomic DNA of *L. reuteri* 100-23 using primers *gadB*-KO1-*Pst*I (5’-AACTGCAGGTTCAATTTTCAGCACATG-3’) and *gadB*-KO2-*Xba*I (5’- GCTCTAGATATCCTGCCATAGATAAAACCTC-3’). The amplicon was ligated into pGEMTeasy vector (Promega) to generate pGadB-A. The plasmid pGadB-B containing the 3’ flanking fragment of *gadB* was created using *gadB*-KO3-*Xba*I (5’- GCTCTAGATTCACTCATTAAACCTTAGAA-3’) and *gadB*-KO4-*Bam*HI (5’- CGGGATCCAATGGCTGCAGGGATA-3’). The flanking fragments of *gadB* from these plasmids were digested with the respective restriction enzymes, purified, and ligated to create pGadB-AB. The DNA fragment in pGadB-AB was cut with *Pst*I and *Bam*HI and ligated into the *Pst*I-*Bam*HI sites in pJRS233. The resulting plasmid pKO-*gadB*-AB was electrotransformed in competent *L. reuteri* 100-23 cells resuspended in water with 30% (v/v) polyethylene glycol (MW 3350; J.T. Baker Chemical, Phillipsburg, NJ). Transformants were grown in mMRS-erythromycin broth at 42 – 44°C for 80 generations to select for single crossover mutants. *L. reuteri* with pKO-*gadB*-AB integrated into chromosome were cured by culturing in mMRS broth at 37°C for 100 generations. The culture was plated on mMRS agar and erythromycin-sensitive double crossover mutants were identified by replica plating mMRS and mMRS-erythromycin agar. The truncation of *gadB* in *L. reuteri* 100-23∆*gadB* was confirmed by PCR with the primers *gadB*-KO1-*Pst*I and *gadB*-KO4-*Bam*HI. An amplicon with the expected size of 3000 bp was obtained with DNA from the *L. reuteri* 100-23 as template. PCR with DNA from *L. reuteri* 100-23∆*gadB* yielded a 2000 bp amplicon. A second PCR with primers *gadB*-5F (5’-GGTCTTATTACCGTTCCTAAT-3’) and *gadB*-6R (5’-ACATTTCTTATGGGATTGCAT-3’) yielded 1700 bp and 500 bp amplicons from the *L. reuteri* 100-23 and *L. reuteri* Δ*gadB*, respectively. DNA sequencing was conducted to verify the deletion region using primers *gadB*-5F and *gadB*-6R.

### Growth in mMRS and survival at pH 2.5

Growth of *L. reuteri* 100-23 and *L. reuteri* Δ*gadB* was assessed in mMRS (pH 6.7) and mMRS acidified to pH values of 4.7 and 3.8. Media were inoculated with overnight cultures, incubated at 37°C, and growth was monitored by measuring the optical density (OD) at 600 nm. To evaluate acid resistance, overnight grown cultures were harvested by centrifugation, washed in 50 mM Na_2_HPO_4_ buffer (pH 7), and resuspended in 50 mM potassium phosphate buffer (pH 2.5) to an OD_600nm_ of 1.0. To assess the contribution of amino acid metabolism to acid resistance, parallel experiments were conducted in potassium phosphate buffer (pH 2.5) supplemented with 10 mM glutamate or arginine. A pH of 2.5 adjusted with HCl was chosen to match conditions previously used to determine the effect of glutamate decarboxylase on acid resistance in *E. coli* and *L. monocytogenes*[10,19,20]. Samples were taken after 0, 1, 3, 5, 8 and 24 h of incubation at pH 2.5 for quantification of amino acids (see below), and to monitor bacterial survival. For determination of bacterial survival, samples were immediately mixed with phosphate buffered saline (PBS, 137 mmol L^-1^ NaCl, 2.7 mmol L^-1^ KCl, 10 mmol L^–1^ Na_2_HPO_4_, and 2 mmol L^-1^ KH_2_PO_4_, pH 8.0) and diluted in PBS buffer prior to plating on MRS agar. For the samples of 24 h acid treatment, 10 mL of culture was centrifuged, resuspended in PBS buffer and plated on MRS agar to determine the bacterial survival. Growth curves and survival at pH 2.5 were determined in triplicate independent experiments.

### Sourdough fermentations and sampling

Sourdough fermentations were performed with *L. reuteri* 100-23 and *L. reuteri* ∆*gadB* to examine the effect of glutamate decarboxylase on growth, acidification, pH, and amino acid concentrations. Doughs were prepared from 40 g whole wheat flour and sterile tap water to achieve a dough yield of 200 [(dough mass/flour mass) × 100], and inoculated at 37°C with an initial cell count of 1 ± 0.5 × 10^7^ CFU g^-1^. Initially, sourdoughs were inoculated with *L. reuteri* 100-23 or *L. reuteri* ∆*gadB* separately, and samples were collected after 0, 6, 12, 24, 48, 72, and 96 h of fermentation. In a second experiment, *L. reuteri* 100-23 and Δ*gadB* were co-cultured in the same sourdough. Doughs were fermented for 96 h (trial I), or for 240 h by backslopping every 48 h (trial II) to match conditions known to select for *L. reuteri*[8]. At each refreshment step, 5% of the ripe sourdough was used as an inoculum for the subsequent fermentation. Samples were taken to measure pH and viable cell counts. The dough samples were stored at -20°C for subsequent DNA extraction and amino acid analysis (see below). Sourdough fermentations were carried out in two independent experiments, and each sample was analyzed in duplicate.

### Quantification of amino acids by HPLC

Cells from phosphate buffer (pH 2.5) were removed by centrifugation. The supernatant (1 vol) was mixed with deionized water (4 vol), saturated potassium borate (4 vol) and β-aminobutyric acid used as internal standard (1 vol). Sourdough samples were lyophilized, extracted with water at an extraction ratio of 1:6 (w/v) and diluted with water, potassium borate, and internal standard as described above. Amino acids were quantified by HPLC after derivatisation with *o*-phtaldialdehyde [31].

### Extraction of DNA from sourdough

Two grams of sourdough were washed with 4 ml of PBS buffer (pH 7.8) and resuspended in PBS. Flour solids were removed by 1500 × *g* for 10 min and cells were harvested by centrifugation at 13,000 × *g*. Cells were resuspended in 660 µL lysis buffer (10 mmol L^-1^ Tris-Cl, 0.5 mmol L^-1^ EDTA, 10% SDS, pH 7.5), and incubated at 65°C for 10 min. Protein was precipitated by addition of 340 µL 5 mol L^-1^ potassium acetate and incubation at -20°C for 10 min. After centrifugation, 900 µL of the supernatant was mixed with an equal volume of isopropanol, DNA was pelleted by centrifugation and washed with 95% ice-cold ethanol. DNA was resuspended in 20 µL of 10 mmol L^-1^ Tris-Cl (pH 8.5), and the DNA concentration was determined by UV spectrometry using a NanoDrop spectrophotometer ND-1000 (Thermo Fisher Scientific Inc., Wilmington, DE, USA).

### Quantification of bacterial population in sourdough using quantitative PCR (qPCR)

The cell counts of *L. reuteri* 100-23 relative to the *L. reuteri* Δ*gadB* was quantified using a probe-based qPCR assay targeting the native and disrupted regions of *gadB*, respectively. Two μg of chromosomal DNA of *L. reuteri* 100-23 and *L. reuteri* Δ*gadB* were digested separately with *Not*I, purified and diluted to a known DNA concentration corresponding to a known copy number *gadB* or ∆*gadB* for use as standards. DNAs extracted from sourdough were also digested with *Not*I and purified using the QIAquick PCR Purification kit (Qiagen). Strain-specific primers and probes were designed using Primer Express Software 3.0 (Applied Biosystems, Streetsville, Ontario, Canada). Primers and probe specific for *L. reuteri* 100-23 (WT-qPCR-F, 5’-TGGGATTTCCAACTAAAGAATGTTG-3’; WT-qPCR-R, 5’-CAACCAATACCAGGATAAACTAAACCA-3’; and WT-qPCR-probe, 5’-Hex/TCCATTAACGCTTCCGGCCACAAGT/IABkFQ-3’) target the DNA region of gene *gadB*, which is deleted in *L. reuteri* Δ*gadB* strain. Primers and probe specific for *L. reuteri* Δ*gadB* (Δ*gadB*-qPCR-F, 5’-CATATCATTGCAAATTCAGACGAAA-3’; Δ*gadB*-qPCR-R, 5’-ATCTAAGCAAGTTGTTATGCTTGTTTAGATC-3’; and Δ*gadB*-qPCR-probe (5’-TET/CCTAGGAGGTTTTATCTATGGCAGGATAATCTAGATTCAC/IABkFQ-3’) target the joint site of deleted *gadB* sequence which is absent in the wild type strain. PCR was carried out with Rox Brilliant II Master Mix (Stratagene, Mississauga, Ontario, Canada) in a 7500 Fast Real-time PCR instrument (Applied Biosystems). Standard curve, determination of the PCR efficiency, and calculation of the copy number were carried out with 7500 Fast System SDS v1.4 software.

### Nucleotide accession numbers

DNA sequences of *L. reuteri* 100-23 were obtained from the National Center for Biotechnology Information databases (GenBank: AAPZ02000002.1). The gene regions of predicted glutamate/γ-aminobutyrate antiporter (*gadC2*), glutaminase (*gls3*), glutamate/γ-aminobutyrate antiporter (*gadC1*), glutamate decarboxylase (*gadB*) and xanthine/uracil/vitamin C permease are 549316..550869, complement (548219..549139), complement (546271..547809), complement (544847..546253) and 543444..544787, respectively. The sequence of the truncated *gadB* in *L. reuteri* Δ*gadB* was deposited with accession number JF339969.

## Results

### Gene locus coding for glutamate metabolism in *L. reuteri*

**Figure 1:**
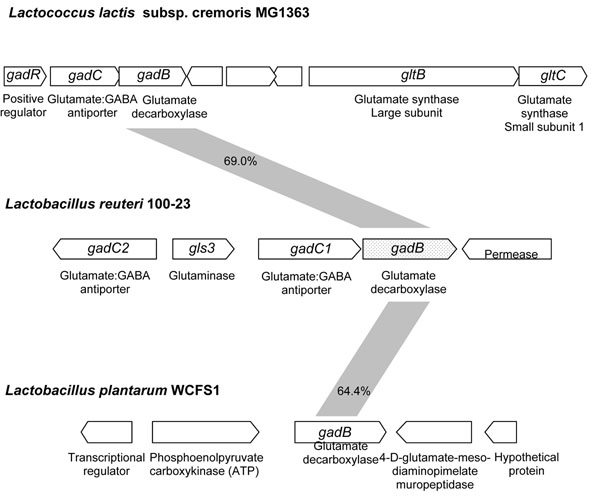
Representation of gene loci encoding glutamate decarboxylases in *Lactococcus lactis* MG1363, *Lactobacillus reuteri* 100-23 and *Lactobacillus plantarum* WCFS1. Numbers indicate protein similarity.

Genome analysis of *L. reuteri* 100-23 revealed a gene cluster encoding proteins homologous to glutamate decarboxylase (*gadB*), glutamate: γ–aminobutyric acid (GABA) antiporters (*gadC1* and *gadC2*), a glutaminase (*gls3*), and a xanthine/uracil/vitamin C permease (Fig. 1). The GadB sequence is 69%, 64.4%, 63.7%, and 51.1% similar to that of *L. lactis* MG1363 [21], *L. plantarum* WCFS1 [28], *L. brevis* OPK-3 [32], and *L. paracasei*[*24*], respectively, suggesting that *gadB* of *L. reuteri* 100-23 encodes a glutamate decarboxylase. However, the organisation of the *gadB* gene locus in *L. reuteri* differs from related lactic acid bacteria as it also contains one of three glutaminases encoded in the genome of *L. reuteri* 100-23. In *Lactococcus lactis* subsp. *cremoris* MG1363, *gadB* is accompanied by *gadC* and *gadR*, which is a positive regulator of *gadBC*, and is adjacent to the glutamate synthase domains *gltB* and *gltC*. In *L. plantarum* WCFS1, *gadB* is not located adjacent to other genes related to glutamine or glutamate metabolism.

### Creation of a *gadB* deletion mutation in *L. reuteri*

**Figure 2:**
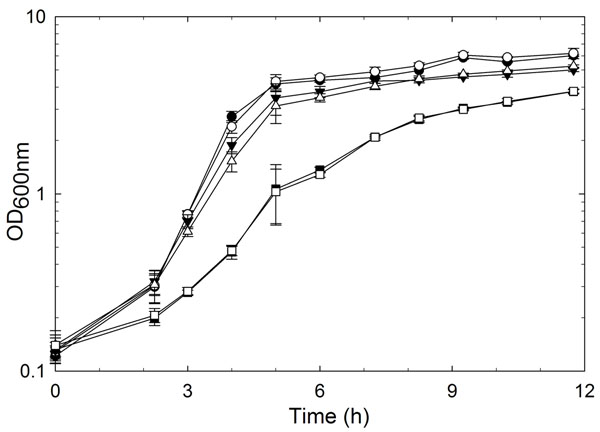
Bacterial growth curve of *L. reuteri* 100-23 (black symbols) and Δ*gadB* (open symbols) strains in mMRS (pH 6.7, ●, ○) and HCl-acidified mMRS (pH 4.7, ▼, ∆; pH 3.8, ■, □).

A deletion in *gadB* of *L. reuteri* 100-23 was generated by a double crossover mutagenesis method. The deletion in the resulting strain *L. reuteri* 100-23 Δ*gadB* was confirmed by DNA sequencing. The impact of *gadB* mutation on bacterial growth was investigated in mMRS and acidified mMRS (Fig. 2). Wild-type *L. reuteri* and the Δ*gadB* mutant grew similarly in mMRS (pH 6.7, 4.7 and 3.8), indicating that the growth of *L. reuteri* Δ*gadB* in complex media was not affected.

### Survival of *L. reuteri* Δ*gadB* at pH 2.5

**Figure 3:**
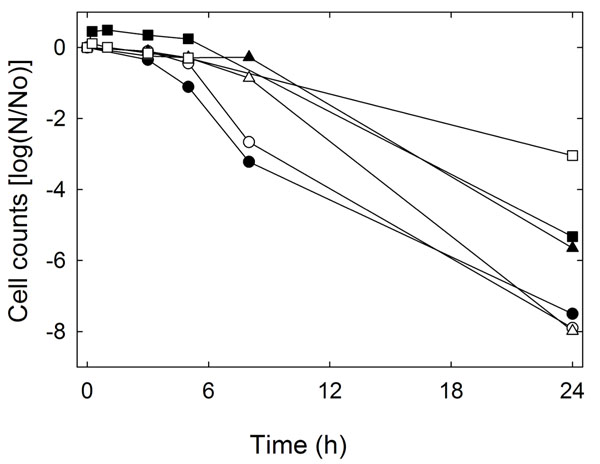
Acid resistance of *L. reuteri* 100-23 (black symbols) and *L. reuteri* Δ*gadB* (open symbols) in phosphate buffer (pH 2.5) (●, ○), in phosphate buffer with 10 mM glutamate (▲, ∆), or in phosphate buffer with 10 mM arginine (■, □). Cell counts are plotted as log (N/N_0_). Data are representative of three independent experiments and the standard deviations were generally less than 0.3.

To confirm that *gadB* of *L. reuteri* encoded glutamate decarboxylase contributes to acid resistance, amino acid metabolism and survival at pH 2.5 of *L. reuteri* ∆*gadB* were compared to the wild type strain *L. reuteri* 100-23. Disruption of *gadB* did not influence the survival of *L. reuteri* at pH 2.5 in the absence of glutamate (Fig. 3). However, addition of glutamate increased survival of *L. reuteri* 100-23 by more than 2 log whereas only a transient effect on survival of *L. reuteri* Δ*gadB* was observed (Fig. 3). Amino acid analysis confirmed that *L. reuteri* 100-23 but not *L. reuteri* Δ*gadB* converted glutamate to GABA. After 24 h of incubation of *L. reuteri* 100-23 in phosphate buffer in presence or absence of glutamate, GABA concentration was 2.5 ± 0.1 and 0.4 ± 0.03 mmol L^-1^, respectively, whereas GABA was not detected in supernatant after incubation of *L. reuteri* Δ*gadB* in presence or absence of glutamate. Taken together, these results demonstrate that *gadB* in *L. reuteri* encodes a glutamate decarboxylase which contributes to acid resistance at pH 2.5.

The effect of glutamate on acid resistance of *L. reuteri* 100-23 and *L. reuteri* ∆*gadB* was compared to the effect of arginine. The effect of arginine on survival of *L. reuteri* 100-23 was comparable to the effect of glutamate (Fig. 3). Notably, *L. reuteri* Δ*gadB* was more resistant in arginine-containing medium than the wild-type strain, indicating that the loss of the function of acid resistance may be compensated by over-expression of alternative pathways to achieve pH homeostasis.

### Glutamine metabolism of *L. reuteri* Δ*gadB* in sourdough

**Figure 4:**
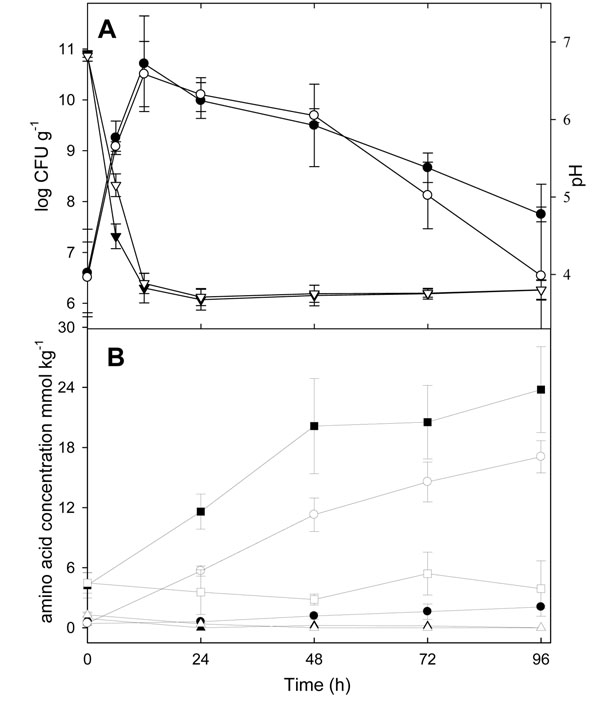
(A) Cell counts (●, ○) and pH (▼, ▽) of sourdough fermented with *L. reuteri* 100-23 (black symbols) or *L. reuteri* Δ*gadB* (open symbols) over 96 h. (B) Concentrations of glutamine (▲, ∆), glutamate (●, ○) and γ-aminobutyric acid (■, □) during sourdough fermentation over 96 h in *L. reuteri* 100-23 (black symbols) and *L. reuteri* Δ*gadB* (open symbols). Symbols indicate means ± standard deviation from quadruplicate determinations.

Because *in vitro* acid challenge demonstrated a contribution of GadB to bacterial survival under acidic conditions, stationary phase survival of *L. reuteri* ∆*gadB* in sourdough was compared to the wild type strain (Fig. 4A). Within 12 h of fermentation, the pH decreased from 6.6 to 3.8. Growth of the wild-type and Δ*gadB* strains in sourdough was virtually identical, maximum cell counts of 10.7 ± 0.9 and 10.5 ± 0.6 log CFU g^-1^, respectively, were reached after 12 h. At 96 h after fermentation, the pH was maintained at 3.8, and the cell counts decreased to 6.5 - 7.7 log CFU g^-1^. Cell counts of *L. reuteri* ∆*gadB* in sourdough consistently decreased faster than cell counts of the wild type strain. A comparable trend towards improved survival was observed when a GadB positive wild type strain was compared to a GadB negative wild type strain [26], however, in both cases, the difference was smaller than experimental error [Fig. 4A and [26]].

The evaluation of glutamine and glutamate metabolism by *L. reuteri* 100-23 and *L. reuteri* ∆*gadB* in sourdough revealed that disruption of *gadB* had no influence on the concentration of total amino acids or the concentration of (glutamine + glutamate + GABA). After 96 h of incubation, the concentration of total amino acids for the wild type and ∆*gadB* strains were 141 ± 7 and 129 ± 8 mmol kg^-1^ DM, respectively. The concentrations of (glutamine + glutamate + GABA) for the wild type and ∆*gadB* strains were 26 ± 4 and 21 ± 4 mmol kg^-1^ DM, respectively. In sourdoughs fermented with both strains, glutamine levels remained low through out fermentation, indicating a quantitative conversion by *L. reuteri* (Fig. 4B). *L. reuteri* 100-23 converted glutamine to GABA, which accumulated to 25 mmol kg^-1^ DM after 96 h of fermentation (Fig. 4B). In contrast, *L. reuteri* ∆*gadB* accumulated glutamate. Low levels of GABA at the beginning of the fermentation and in the sourdoughs fermented with *L. reuteri* ∆*gadB* are attributable to interference by tyrosine, whose retention time was close to that of GABA.

### Role of *gadB* in long-term sourdough fermentation

**Figure 5:**
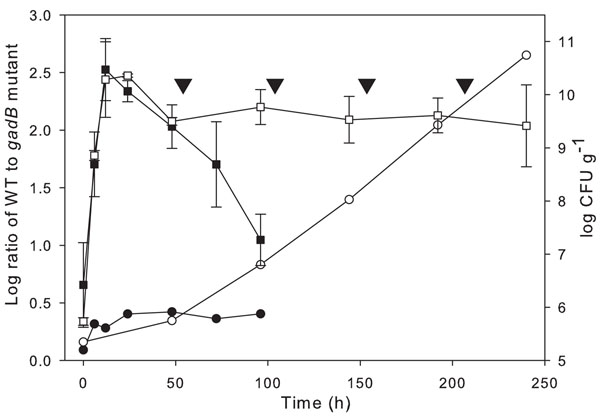
Kinetics of bacterial population in type II sourdough fermentations. Total bacterial cell counts (■,□) were enumerated by plating on mMRS agar. The relative quantification of wild type and mutant strains was achieved by qPCR (●,○). Sourdoughs were fermented in a single batch over 96 h (trial I; ■, ●), or over 240 h with refreshment (back-slopping with 5% inoculum) every 48 h (trial II; □, ○). The time points of refreshment are indicated (▽). Symbols indicate means ± standard deviation from duplicate independent experiments analysed in duplicate. The standard deviations of cell copy number ratio were less than 0.2 log.

The disruption of *gadB* did not significantly influence survival of *L. reuteri* over 96 h of fermentation. However, in sourdoughs maintained by continuous backslopping, relatively small differences between cell counts in a single batch will accumulate over subsequent fermentation cycles. Therefore, it was investigated whether *gadB* disruption affects the ecological fitness of *L. reuteri* ∆*gadB* in competition with the wild type strain in a sourdough that was backslopped every 48 h. A sourdough fermented over 96 h without backslopping was used for comparison. In sourdough without backslopping, the growth of the co-culture was similar to that of single strains (Fig. 4A and Fig. 5). The relative cell counts of *L. reuteri* 100-23 and *L. reuteri* ∆*gadB* were determined by qPCR using strain-specific primers and probes. Cell counts of *L. reuteri* 100-23 relative to *L. reuteri* ∆*gadB* increased slightly from 1.3 to 2.7 log during 96 h of incubation, suggesting that disruption of *gadB* slightly compromised stationary phase survival. In the backslopped sourdoughs, the relative cell counts of *L. reuteri* 100-23 to *L. reuteri* ∆*gadB* also increased from about 1.5 to 2 log during the first fermentation cycle. With every subsequent fermentation cycle, the log ratio (wild type/∆*gadB*) increased by about 0.5 (Fig. 5). At the end of the fifth fermentation cycle, cell counts of *L. reuteri* 100-23 relative to *L. reuteri* ∆*gadB* were 460:1, demonstrating that *gadB* in *L. reuteri* is essential for its competitiveness in type II sourdoughs maintained by continuous propagation.

## Discussion

This study investigated the role of *gadB* coding for glutamate decarboxylase in the acid resistance of *L. reuteri*, and demonstrated that glutamate decarboxylation contributes to the competitiveness of *L. reuteri* in type II sourdoughs that are propagated by continuous backslopping.

The contribution of metabolic traits of *L. reuteri* to its competitiveness in the intestine was previously evaluated with insertional mutations generated by single-crossover mutagenesis [6,33]. This study employed a double crossover method to generate an in-frame deletion of *gadB* in *L. reuteri* 100-23. This approach avoids interference by antibiotic-resistance genes or other plasmid-borne foreign genes that remain on the chromosome of mutant strains generated by single-crossover mutagenesis.

The identification of lactic acid bacteria in sourdoughs was previously determined by using species-specific, semi-quantitative denaturing gradient gel electrophoresis (DGGE) [8,9,34,35] or culture-dependent enumeration, followed by strain identification through random amplified polymorphic DNA [34,36]. However, culture-dependent enumeration or DGGE fails to account for species or strains that contribute less than 1% to the total cell counts. The qPCR method used in this study achieved an accurate and consistent relative quantification of *L. reuteri* 100-23 and *L. reuteri* ∆*gadB* even in sourdoughs in which *L. reuteri* ∆*gadB* contributed less than 0.5% of the total population.

**Figure 6:**
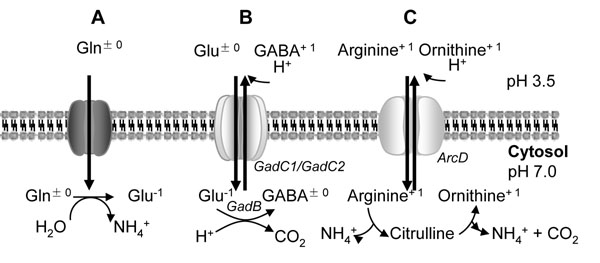
Amino-acid based acid resistance mechanisms in *L. reuteri*. Following uptake of glutamine by unknown mechanisms, glutaminase catalyzes deamidation to glutamate and ammonia (Fig. 6A). Glutamate is taken up by the electrogenic glutamate/γ;-aminobutyric acid (GABA) antiport system. Decarboxylation of glutamate consumes an intracellular proton and contributes to generation of Δ;Ψ; and Δ; pH. Extracellular protonation of GABA consumes additional protons (Fig. 6B). Arginine is taken up by electroneutral arginine/ornithine exchange. Ammonia generated from arginine contributes to intracellular pH homeostasis; intra- or extracellular protonation of ornithine consumes additional protons (Fig. 6C). GadB: glutamate decarboxylase; GadC: putative glutamate:GABA antiporter; ArcD: putative arginine:ornithine antiporter. Relevant pK_A_ values of amino acid side chains and GABA are: glutamate, 4.25; GABA, 4.23; arginine, 12.5; ornithine, 8.69.

Type II sourdough fermentations are characterized by a short period of growth, followed by an extended period of fermentation at pH 3.2 – 3.6 [3,4]. In long-term sourdough fermentations, cereal proteases continuously supply amino acids from cereal proteins, and thus support bacterial acid resistance based on amino acid metabolism [17,18]. Arginine and glutamate often contribute to acid resistance of Gram-negative and Gram-positive bacteria (Fig. 6) [10,15,37].

The arginine deiminase (ADI) pathway generates ATP, consumes intracellular protons, and causes the alkalization of the fermentation substrate [Fig. 6; [10,11,15]]. In keeping with prior studies, arginine improved the survival of *L. reuteri* at pH 2.5 about 10-100 fold [[16], this study]. Glutamate decarboxylase was characterized in *L. brevis* and *L. paracasei*[23,24], and GadB-positive lactobacilli were employed to accumulate GABA as a functional food ingredient in food fermentations [26,38]. The strain-dependent expression of glutamate decarboxylase in lactobacilli, particularly *L. reuteri*, indicates that GadB is not essential for growth [26]. This was confirmed by comparison of the growth of *L. reuteri* 100-23 and the ∆*gadB* mutant strain in mMRS and sourdough (Figs. 2, 4 and 5). However, although glutamate decarboxylation generally contributes to bacterial resistance to acid [10,15], its contribution to acid resistance in acid-tolerant lactobacilli has not been demonstrated experimentally [26]. Glutamate decarboxylation is particularly effective at pH values below the pK_A_ of GABA, 4.23, as protonation of GABA below the pK_A_ results in alkalization of the external fermentation substrate (Fig. 6). Accordingly, this study demonstrated a protective effect of glutamate decarboxylation at pH 2.5 in buffer, and at pH 3.6 in sourdough. Sourdough is acidified by lactic and acetic acids rather than HCl. However, growth and survival of lactobacilli in sourdough is determined by the pH and not by the concentration of undissociated organic acids [39]. The protective effect of glutamate on the survival of *L. reuteri* at pH 2.5, an about 100-fold increase of cell counts after acid challenge, is comparable to the effect in *E. coli*[19,37]. In *L. monocytogenes*, glutamate addition improved survival after exposure pH 2.5 of a wild type strain more than 6 log compared to a *gadAB* deficient mutant [20].

A contribution of glutamine deamidation to bacterial acid resistance has not been demonstrated [10,15,40] but glutamine improved the growth of *L. reuteri* and *L. sanfranciscensis* at low pH [18]. Glutamine accounts for about 30% of the amino acids wheat proteins but the amount of glutamate is low [41]. Consequently, proteolysis in wheat dough liberates glutamine rather than glutamate [18,26]. Glutamate-mediated acid resistance in sourdough thus depends on the conversion of glutamine to glutamate. *L. reuteri* 100-23 harbours three putative glutaminase genes and *gls3* is located adjacent to *gadB*. The substrate availability in cereal fermentations and the arrangement of gene loci strongly indicates that the glutamate-based acid resistance of *L. reuteri* 100-23 uses glutamine as a substrate (Fig. 6). In contrast, *L. lactis* MG1363 has genes coding for glutamine synthase downstream of the *gadB*C operon, and in *L. plantarum* WCFS1, no genes related to glutamine metabolism are located close to the *gadB* (Fig. 1). The exceptional arrangement of glutamine / glutamate catabolic genes in *L. reuteri* may reflect the adaptation to cereal substrates. Glutamine:GABA antiport by *gadC* or analogues has not been shown experimentally [22]. However, ATP-dependent glutamate transport enzymes in *L. lactis* and *L. delbrueckii* also transport glutamine with high affinity [42,43].

The disruption of *gadB* did not influence the survival of *L. reuteri* during long-term sourdough fermentation unless the sourdough was backslopped. The loss of one metabolic pathway for pH homeostasis in *L. reuteri* is apparently partially compensated by alternative pathways (Fig. 6). However, monitoring of the microbiota in sourdoughs maintained by continuous backslopping is considered the most appropriate tool to identify competitive strains [8,34-36]. The observation that the wild type strain displaced *L. reuteri* ∆*gadB* after only a few refreshments demonstrates that acid resistance in general and glutamate decarboxylase in particular contributes to the competitiveness of *L. reuteri* in type II sourdoughs.

In addition to its occurrence in sourdough fermentations, *L. reuteri* is recognized as a stable member of the intestinal microbiota of humans and animals, and strains in the species have evolved to colonize the gastrointestinal tracts of specific hosts [6]. In pigs, poultry, and rodents, *L. reuteri* colonizes the pars oesophagus, the crop, and the forestomach, respectively [44]. It is interesting to note that sourdough isolates but not human or poultry isolates of *L. reuteri* are able to colonize rodents [45,46]. Colonization and biofilm formation on the stratified, squamous epithelia lining the proximal gastrointestinal tract of animals occurs upstream of the stomach [44,45]. Therefore, intestinal *L. reuteri* are exposed to stomach acidity. The conversion of glutamate to GABA in *L. reuteri* is strain-specific but was reported in human, rodent, and sourdough isolates [This study, [26]]. Mechanisms of acid resistance that are relevant in sourdough thus likely contribute to the competitiveness of *L. reuteri* in intestinal ecosystems, and may improve gastro-intestinal survival of *L. reuteri* commercially used as probiotics.

## Conclusions

This study demonstrated that the conversion of glutamate to GABA by *L. reuteri* 100-23 contributes to acid resistance and to competitiveness in type II sourdough fermentations. The organization of the gene cluster for glutamate conversion, and the availability of amino acids in cereals imply that glutamine rather than glutamate functions as the substrate for GABA formation. The exceptional coupling of glutamine deamidation to glutamate decarboxylation in *L.Â reuteri* may reflect ecological adaptation to cereals, or to the proximal intestinal tracts of animals that predominantly feed on cereal grains.

## List of abbreviations used

MRS: deMan-Rogosa-Sharpe medium; LB: Luria-Bertani broth; GABA: γ-aminobutyrate; HPLC: high performance liquid chromatography; PBS: phosphate buffered saline; qPCR: quantitative real time polymerase chain reaction.

## Competing interests

The authors declare that they have no competing interests.

## Authors' contributions

MGG initiated and coordinated the project; MSS and SS planned and performed the experiments. MSS and MGG wrote the paper. All authors approved the final version of the manuscript.
